# 

**Published:** 1918-03-02

**Authors:** 


### CONTENTS.

PAGE

**The General Practitioner and Consumption...** 449

**Man-Power and Men with Defective Physique** 450

**Hospital and Institutional News ... ... ...** 451
The Prince of Wales and King Edward VII.'s Hospital—An Interesting Guy's Legacy—Secretaries and Salaries—Portuguese Patients at Exeter—The English Cottage Hospital—The Staff Record of Sunderland Royal Infirmary—A Table of Workmen's Subscriptions—A New "County Hospital" at Brighton—Women Hospital Secretaries—Montgomery Hospital's Jubilee—Progress at Aberavon—Further Extension of the Third Western General Hospital—A Census of Limbless Cases—A Museum's War Record—"Kitchener House"—"The Bath Bun"—The *Southern Cross* in Eclipse—Church Collections for Hospital Sunday—This Week's Drug Market.

**Papers on Madness ... ... ... ... ... ...** 455

I. Madhouses before A.D. 1846,

**Of Medical Certificates ... ... ... ... ...** 456

**Lord Lister ... ... ... ... ... ... ...** 457

VI. Lister's Hypothesis of the Cause of Suppuration.

**Hospitals in the Holy Land ... ... ...** 461

I. Church Missions Hospital, Jerusalem.

**Nursing Affairs in Ireland ... ... ... ...** 462

Camouflage.

PAGE

**The Institutional Library ... ... ... ...** 459
Syphilis and the Army—Serums, Vaccines, and Toxins—Hygiene and Public Health—Modern Methods in Nursing—Health of the Munition Worker—Modern Dietetics—The Medical Register—Our Girls in War Time.

**The Matrons' and Sisters' Department ...** 463
Nursing Progress and its Developments: VIII. Supporters of the College.
Round the Hospitals.
Two College Centres Inaugurated—Sir Arthur Stanley and the Critics—Miss Cummins' Courageous Speech—Camouflage in Nursing Politics—"Ierne's" Interesting Challenge—Why Matrons Must Exhibit Courage.

**Food Problem Notes ... ... ... ... ... ...** 466

**The College of Nursing ... ... ... ...** 467

Liverpool Centre Inaugurated.

**Matrons' and Sisters' Appointments ... ...** 472

**The Editor's Letter-Box ... ... ... ...** 472

Tact in Sanatorium Management.

**Recently Published Parliamentary Returns, etc.** 472

**The Institutional Worker ... ... (Suppl.)** 1
Extending a Hospital Engineer's Work: Answer to January Question.
Crockery Washing in a Nurses' Home: Answer to January Question.

